# Sanguinaria Nit

**Published:** 1885-07

**Authors:** J. S. Smith


### SANGUINARIA NIT.

Mrs. N., æt. forty-three. Change of life. Suffered more or less constantly with dull pain and burning in pit of stomach and left ovarian region. Better from hard pressure, but forcing her to loosen her clothes, and relieved by food for from ten minutes to several hours. General health good. Under treatment off and on for several months. Nothing given affording any permanent relief. In the fall of 1883 she was given Sang. nit. 3x for severe coryza, which was at once relieved, and with it the distressing symptoms which had proved so intractable, and which did not return for several months, when one or two powders again relieved with no further trouble till August, 1884. One powder again did the work, with no return since. The cure was purely accidental and the drug was not given to that end. But as the provings we have of it are brief and imperfect, the above is recorded as a clinical fact which may possibly prove useful. J. S. SMITH, M. D.

